# C‐reactive protein level as a predictor of difficult emergency laparoscopic cholecystectomy

**DOI:** 10.1002/bjs5.50189

**Published:** 2019-07-02

**Authors:** H. J. Ng, Z. Ahmed, K. S. Khan, T. Katbeh, A. H. M. Nassar

**Affiliations:** ^1^ Department of General Surgery University Hospital Monklands, NHS Lanarkshire Airdrie ML6 0JS UK

## Abstract

**Background:**

Studies focused on C‐reactive protein (CRP) as a marker of difficult laparoscopic cholecystectomy are limited to small case series. The aim of this study was to evaluate the association between preoperative CRP concentration and difficulty of laparoscopic cholecystectomy in patients admitted with a biliary emergency presentation.

**Methods:**

Patients with an emergency admission for biliary disease treated between 2012 and 2017 with a documented preoperative CRP level were analysed. Elective patients and those with other concurrent causes of increased CRP concentration were excluded. The intraoperative difficulty grade was based on the Nassar scale. Statistical analysis was conducted to determine the association of preoperative CRP level with difficulty grading, adjusted for the interval to surgery.

**Results:**

A total of 804 emergency patients were included. The mean preoperative peak CRP level was 64·7 mg/l for operative difficulty grade I, 69·6 mg/l for grade II, 98·2 mg/l for grade III, 217·5 mg/l for grade IV and 193·1 mg/l for grade V, indicating a significant association between CRP concentration and Nassar grade (*P* < 0·001). Receiver operating characteristic (ROC) curve analysis showed an area under the curve of 0·78 (95 per cent c.i. 0·75 to 0·82), differentiating patients with grade I–III from those with grade IV–V operative difficulty. ROC curve analysis found a cut‐off CRP value of 90 mg/l, with 71·5 per cent sensitivity and 70·5 per cent specificity in predicting operative difficulty of grade IV or V. Logistic regression analysis found preoperative peak CRP level to be predictive of Nassar grade I–III *versus* grade IV–V operative difficulty, also when adjusted for timing of surgery (odds ratio 5·90, 95 per cent c.i. 2·80 to 12·50).

**Conclusion:**

Raised preoperative CRP levels are associated with greater operative difficulty based on Nassar scale grading.

## Introduction

The Japanese Society of Hepato‐Biliary‐Pancreatic Surgery developed the Tokyo Guidelines in 2007 to diagnose and grade the severity of acute cholecystitis based on local clinical signs (Murphy's sign, right upper quadrant mass, pain and gallbladder tenderness), systemic signs of inflammation (fever, raised C‐reactive protein (CRP) level and increased white cell count (WCC)) and imaging findings^1^.

Acute cholecystitis is a well recognized cause of difficult laparoscopic treatment. A number of studies have documented that CRP concentration is a good predictor of acute cholecystitis^2^, ^3^ with better discriminative power than WCC^4^. Studies^5^, ^6^, ^7^, ^8^ have also correlated CRP level with the conversion rate of laparoscopic cholecystectomy, considering conversion as a surrogate of laparoscopic difficulty.

Traditional management of acute cholecystitis was based on conservative treatment with antibiotics and analgesics during the acute phase, followed by delayed cholecystectomy after a number of weeks. However, other authors^9^, ^10^ have recently suggested that patients admitted acutely with right upper‐quadrant pain suggestive of biliary origin with documented gallstones on ultrasound imaging could be scheduled for immediate laparoscopic cholecystectomy if assessed as fit for general anaesthesia and surgery.

The aim of this study was to evaluate the association between preoperative CRP level and operative difficulty of emergency laparoscopic cholecystectomy in acute biliary admissions.

## Methods

All consecutive patients undergoing laparoscopic cholecystectomy performed by a single surgeon or by trainees under direct supervision between September 2012 and December 2017 in a teaching hospital were reviewed. Inclusion criteria for data analysis were: patient with biliary symptoms admitted as an emergency who underwent laparoscopic cholecystectomy and intraoperative cholangiography (IOC) with or without common bile duct (CBD) exploration, and a documented preoperative CRP value. Exclusion criteria were: elective admission and patient with concurrent non‐biliary cause of raised CRP concentration (chest infection).

Data on patient demographics, radiological findings, timing of surgery, duration of surgery, conversion, perioperative complications, readmissions and mortality were retrieved. Surgical difficulty was based on the Nassar scale^11^ and a CRP level below 6 mg/l was considered normal^12^.

### Patient management

According to hospital protocol, all patients with an acute admission diagnosis consistent with benign biliary disease on the basis of clinical and ultrasound findings are referred to the biliary team. Emergency laparoscopic cholecystectomy is performed during the index admission if the patient is assessed as fit for general anaesthesia. Patients are scheduled for a delayed laparoscopic cholecystectomy if unfit for surgery, if they are referred from other centres or if they have a medical history of previous admission for the same clinical reason.

All emergency admissions undergo chest X‐ray and urine analysis to exclude other causes of abdominal pain. Magnetic resonance cholangiopancreatography (MRCP) is not used as part of the diagnostic protocol and endoscopic retrograde cholangiopancreatography (ERCP) is not performed for preoperative removal of bile duct stones, except in patients unfit for general anaesthesia.

CT is performed in cases of suspected malignancy, suspected severe pancreatitis or severe cholecystitis. All patients assessed fit for surgery undergo laparoscopic cholecystectomy with routine IOC and, if necessary, CBD exploration by a dedicated biliary surgical team.

### Statistical analysis

Continuous variables are presented as mean(s.d.) and median (range) values and compared with Student's *t* test. Categorical variables are presented as frequencies with percentages and compared using the χ^2^ test.

Logistic regression analysis was used to assess predictors of high operative difficulty (grade III or above) *versus* low difficulty (grades I and II), and included the following co‐variables: age, sex, interval to surgery and peak CRP level of 90 mg/l or above.

Receiver operating characteristic (ROC) curves were used to determine the accuracy of preoperative peak CRP levels, with values expressed as the area under the curve (AUC) with 95 per cent confidence limits.

*P* < 0·050 was considered statistically significant. All analyses were performed using Stata® release 14 (StataCorp, College Station, Texas, USA).

## Results

**Figure 1:**
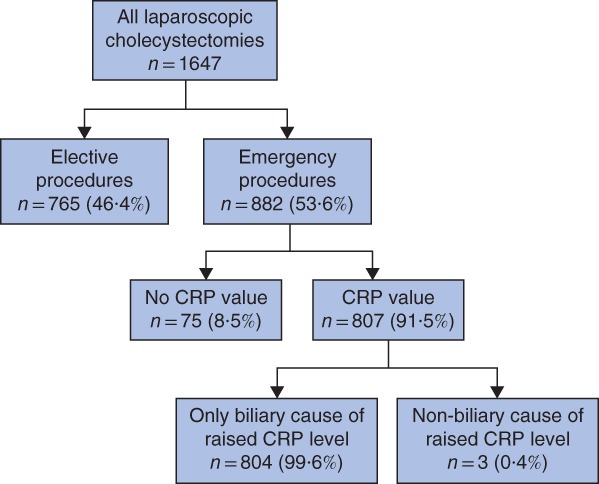
Flow diagram of patient inclusion in the study CRP, C‐reactive protein.

Of 1647 laparoscopic cholecystectomies performed between 2012 and 2017, 882 (53·6 per cent) were performed as an emergency. Some 807 patients had preoperative estimation of CRP concentration, but three were excluded owing to preoperative chest or urinary infection (*Fig*. 1).

Patients' demographic and clinical characteristics are summarized in *Table* 1. The admission diagnosis was biliary colic in 258 of the 804 patients (32·1 per cent), obstructive jaundice in 189 (23·5 per cent), acute cholecystitis in 177 (22·0 per cent), acute biliary pancreatitis in 135 (16·8 per cent) and acute cholangitis in 45 (5·6 per cent). Thirty‐seven patients (4·6 per cent) were referred from other hospitals.

**Table 1:** Patient demographics

	No. of patients* (*n* = 804)
**Age (years)**	
Mean(s.d.)	53·5(17·9)
Median (range)	54·0 (15·0–91·0)
**Sex ratio (M** : **F)**	249 : 555
**ASA fitness grade**	
I	249 (31·0)
II	353 (43·9)
III	173 (21·5)
IV	10 (1·2)
Unknown	19 (2·4)

* With percentages in parentheses unless indicated otherwise.

A medical history of previous admission was recorded for 86 patients (10·7 per cent), with a mean of 1·1 admissions per patient. These included acute biliary pain in 36 patients, and previous episodes of obstructive jaundice in 24 patients, pancreatitis in eight and acute cholecystitis in 18. However, only ten of the 86 patients with a previous history of biliary disease had been treated previously at University Hospital Monklands.

The majority (95·6 per cent) of patients who had laparoscopic cholecystectomy plus IOC, with or without CBD exploration, were evaluated by ultrasound imaging of the abdomen. In six patients (0·7 per cent), only CT was performed. Nine patients (1·1 per cent) were evaluated using MRCP exclusively. Thirty‐nine patients (4·9 per cent) were investigated using both ultrasonography and MRCP, whereas 23 (2·9 per cent) were studied with ultrasound imaging and CT, one patient (0·1 per cent) with CT and MRCP, and seven (0·9 per cent) with all the three modalities (ultrasonography, MRCP and CT). Seventeen patients had ERCP before surgery, and the majority (82·0 per cent) were referred to the biliary service from other hospitals owing to failed ERCP and CBD clearance. Some 604 patients (75·1 per cent) had surgery within 5 days of admission.

A total of 567 patients were treated by a consultant surgeon and 136 (16·9 per cent) by a trainee assisted by the consultant; in the remaining 101 patients (12·6 per cent), trainees performed a few surgical steps with tutorship. The duration of surgical procedure ranged from 22 to 345 min. The median time for laparoscopic cholecystectomy was 75 min, that for laparoscopic cholecystectomy plus IOC was 56 min, and that for laparoscopic cholecystectomy plus IOC with CBD exploration (39·7 per cent) was 90 min.

There were no conversions to open surgery. Duration of hospital stay ranged from 1 to 60 (median 8) days. The complication rate was 3·9 per cent and the 30‐day readmission rate 3 per cent. Only one patient died within 30 days of surgery, following an uneventful laparoscopic cholecystectomy with the IOC showing an unexplained distortion of the left hepatic duct. Further postoperative radiological imaging showed a pseudoaneurysm of a left hepatic artery branch. The patient was transferred to a specialist liver unit to undergo interventional radiology, but died from blood loss following attempted embolization.

No association was found between stratification of Nassar scale grades and the timing of laparoscopic cholecystectomy (*Table* 2) (*P* = 0·415), but patients with a previous admission for biliary disease had increased operative difficulty than those with a first admission (*Table* 3) (*P* < 0·001).

**Table 2:** Nassar scale grade and timing of surgery

Timing of surgery (days)	Grade I (*n* = 180)	Grade II (*n* = 241)	Grade III (*n* = 189)	Grade IV (*n* = 154)	Grade V (*n* = 40)	*P* *
0–1	59 (32·8)	100 (41·5)	69 (36·5)	67 (43·5)	14 (35)	0·415
2–5	74 (41·1)	100 (41·5)	73 (38·6)	56 (36·4)	17 (43)	
6–9	31 (17·2)	28 (11·6)	31 (16·4)	25 (16·2)	5 (13)	
≥ 10	16 (8·9)	13 (5·4)	16 (8·5)	6 (3·9)	4 (10)	

Values in parentheses are percentages.

* χ^2^ test.

**Table 3:** Nassar scale grade for patients with a first admission *versus* those who had a previous admission for biliary disease

Nassar scale grade	First admission (*n* = 718)	Previous admission (*n* = 86)	*P* *
I	170 (23·7)	10 (12)	< 0·001
II	223 (31·1)	18 (21)	
III	165 (23·0)	24 (28)	
IV	134 (18·7)	19 (22)	
V	26 (3·6)	15 (17)	

Values in parentheses are percentages.

* χ^2^ test.

### C‐reactive protein

Some 175 patients (21·8 per cent) had a preoperative peak CRP level of less than 6 mg/l, and 629 patients (78·2 per cent) had a raised CRP concentration (greater than or equal to 6 mg/l). The preoperative peak CRP value was increased in 153 patients with biliary pain, 163 patients admitted for acute cholecystitis, 146 with obstructive jaundice, 126 with acute pancreatitis and 41 with acute cholangitis.

**Figure 2:**
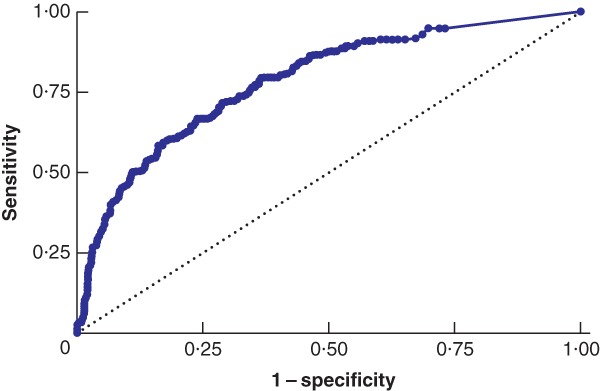
Receiver operating characteristic (ROC) curve analysis of peak preoperative C‐reactive protein levels and its impact on the difficulty grading of intraoperative cholecystectomyNassar scale grade I–III *versus* grade IV–V. Area under the curve 0·78 (95 per cent c.i. 0·75 to 0·82).

The preoperative peak CRP level ranged from less than 6 to 597 mg/l. A statistically significant association was found between the proportion of patients with a CRP level greater than or equal to 6 mg/l and Nassar scale grades (*P* < 0·001) (*Table* 4), and also between the mean peak CRP concentration and Nassar scale grades (*P* < 0·001) (*Table* 5). ROC curve analysis showed an AUC of 0·78 (95 per cent c.i. 0·75 to 0·82) for preoperative CRP as a predictor of operative difficulty grade IV or V (*Fig*. 2). A preoperative CRP cut‐off value of 90 mg/l predicted with 71·5 per cent sensitivity and 70·5 per cent specificity patients who were found to have operative difficulty grade IV or V at laparoscopic cholecystectomy.

**Table 4:** Nassar scale grade and C‐reactive protein subgroups

Nassar scale grade	Peak CRP < 6 mg/l (*n* = 175)	Peak CRP ≥ 6 mg/l (*n* = 629)	*P* *
I	62 (35·4)	118 (18·8)	< 0·001
II	70 (40·0)	171 (27·2)	
III	33 (18·9)	156 (24·8)	
IV	8 (4·6)	145 (23·1)	
V	2 (1·1)	39 (6·2)	

Values in parentheses are percentages. CRP, C‐reactive protein.

* χ^2^ test.

**Table 5:** Nassar scale grade and peak C‐reactive protein levels

Peak CRP (mg/l)	Grade I	Grade II	Grade III	Grade IV	Grade V	*P* *
Mean(s.d.)	64·7(106·4)	69·6(98·4)	98·2(104·1)	217·5(148·5)	193·1(157·6)	< 0·001
Median (range)	14·0 (< 6–597)	20·0 (< 6–508)	65·0 (< 6–523)	226·0 (< 6–578)	145·0 (< 6–523)	

* Student's *t* test.

Logistic regression analysis found preoperative peak CRP concentration to be predictive of Nassar grade I–III *versus* grade IV–V operative difficulty, also when adjusted for days from admission to surgery (OR 5·90, 95 per cent c.i. 2·8 to 12·5) (*Table* 6). This model also demonstrated that each day from admission to surgery reduced the risk of a grade IV/V cholecystectomy (odds ratio (OR) 0·61, 95 per cent c.i. 0·50 to 0·73). Conversely, increasing age at presentation was associated with more difficult procedures, although with a modest OR of 1·03 (1·01 to 1·06) (*Table* 6).

**Table 6:** Logistic regression analysis of predictors of higher operative difficulty (Nassar scale grade IV–V)

	Odds ratio
Peak CRP > 90 mg/ml	5·90 (2·80, 12·50)
No. of days from admission to surgery	0·61 (0·50, 0·73)
Age	1·03 (1·01, 1·06)
Sex (male baseline)	0·58 (0·30, 1·30)

Values in parentheses are 95 per cent confidence intervals. CRP, C‐reactive protein. Likelihood ratio test statistic for the logistic regression model: χ^2^ = 96·1, *P* < 0·001.

## Discussion

Previous studies in this field^2^, ^3^, ^4^ reported an association between a raised CRP level and cholecystitis, although with limited results in relation to duration of symptoms or timing of laboratory test evaluation. Another study^13^ found a CRP concentration above 200 mg/l to be a good predictor of gangrenous cholecystitis, consistent with the present findings and Nassar scale grading.

Several studies found an association between increased CRP levels and difficult laparoscopic cholecystectomy requiring conversion to open surgery, although with different CRP values (ranging from above 5·5 mg/l to 165 mg/l) and different conversion rates reported (range 5–30 per cent)^5^, ^6^, ^7^, ^8^.

In the present series, patients were treated by a dedicated biliary surgical team, with no conversions to open surgery, despite CRP levels as high as 597 mg/l. However, the median hospital stay of 8 days was due to the large percentage of referrals from other departments or hospitals (80·6 per cent of the series), a large proportion of patients having laparoscopic CBD exploration (39·7 per cent) and, occasionally, limited access to emergency theatre sessions.

Some authors have acknowledged the importance of grading the difficulty of cholecystectomy^11^, ^14^ and the use of operative risk predictive scores. The Nassar scale has been suggested to optimize the perioperative management of patients with complicated gallstone disease^14^.

In the present study, the Nassar scale grade was higher when the preoperative peak CRP concentration was raised. An increased CRP level can be a predictor of a high operative difficulty grade, and this finding could be of use in planning surgical management and also to select patients appropriate for surgical training.

Also in this study, as expected, operations with grade I difficulty had the highest percentage of trainees (26·7 per cent) performing the emergency laparoscopic cholecystectomy. As the grade of operative difficulty increased, the consultant was more involved. However, even in Nassar grade V operations trainees were allowed to perform parts of the procedure under the supervision of the consultant.

Possible limitations of this approach relate to logistical difficulties and lack of early access to emergency theatre or a dedicated biliary surgical team; this would impair the large‐scale adoption of this protocol. However, and consistent with the results of the present study, the early identification of potentially complex operations (Nassar grade IV–V) by means of a simple and reliable serological marker could be helpful in planning management and resources.

## Disclosure

The authors declare no conflict of interest.
